# An update on MSA: premotor and non-motor features open a window of opportunities for early diagnosis and intervention

**DOI:** 10.1007/s00415-020-09881-6

**Published:** 2020-05-20

**Authors:** Viorica Chelban, Daniela Catereniuc, Daniela Aftene, Alexandru Gasnas, Ekawat Vichayanrat, Valeria Iodice, Stanislav Groppa, Henry Houlden

**Affiliations:** 1 Department of Neuromuscular Diseases, Queen Square Institute of Neurology, University College London, London, WC1N 3BG UK; 2 Neurobiology and Medical Genetics Laboratory, “Nicolae Testemitanu” State University of Medicine and Pharmacy, 165, Stefan cel Mare si Sfant Boulevard, 2004 Chişinău, Republic of Moldova; 3 Department of Neurology, Epileptology and Internal Diseases, Institute of Emergency Medicine, 1, Toma Ciorba Street, 2004 Chişinău, Republic of Moldova; 4 Department of Neurology nr. 2, Nicolae Testemitanu” State University of Medicine and Pharmacy, 165, Stefan cel Mare si Sfant Boulevard, 2004 Chişinău, Republic of Moldova; 5 Cerebrovascular Diseases and Epilepsy Laboratory, Institute of Emergency Medicine, 1, Toma Ciorba Street, 2004 Chişinău, Republic of Moldova; 6 Autonomic Unit, National Hospital for Neurology and Neurosurgery, UCL NHS Trust, London, WC1N 3BG UK; 7 Neurogenetics Laboratory, National Hospital for Neurology and Neurosurgery, Queen Square, London, WC1N 3BG UK

**Keywords:** Multiple system atrophy, Non-motor features, Premotor phase, MSA

## Abstract

In this review, we describe the wide clinical spectrum of features that can be seen in multiple system atrophy (MSA) with a focus on the premotor phase and the non-motor symptoms providing an up-to-date overview of the current understanding in this fast-growing field. First, we highlight the non-motor features at disease onset when MSA can be indistinguishable from pure autonomic failure or other chronic neurodegenerative conditions. We describe the progression of clinical features to aid the diagnosis of MSA early in the disease course. We go on to describe the levels of diagnostic certainty and we discuss MSA subtypes that do not fit into the current diagnostic criteria, highlighting the complexity of the disease as well as the need for revised diagnostic tools. Second, we describe the pathology, clinical description, and investigations of cardiovascular autonomic failure, urogenital and sexual dysfunction, orthostatic hypotension, and respiratory and REM-sleep behavior disorders, which may precede the motor presentation by months or years. Their presence at presentation, even in the absence of ataxia and parkinsonism, should be regarded as highly suggestive of the premotor phase of MSA. Finally, we discuss how the recognition of the broader spectrum of clinical features of MSA and especially the non-motor features at disease onset represent a window of opportunity for disease-modifying interventions.

## Introduction

Multiple system atrophy (MSA) is a sporadic, adult-onset, progressive, rare neurodegenerative disorder of uncertain etiology. It manifests with diverse clinical features, characterized mainly by a combination of parkinsonism with a poor response to levodopa treatment, cerebellar features, and autonomic failure. The definite diagnosis of MSA can still only be established pathologically with the presence of glial cytoplasmic inclusions (GCI) at postmortem [1–3].

In the past, a variety of terms were used to describe MSA. In 1900, Joseph Jules Dejerine and André Thomas first reported two cases presenting with cerebellar ataxia and pathological lesions in the cerebellum and brainstem, described as olivopontocerebellar atrophy. Sixty years later, Shy and Drager described two patients with parkinsonism and autonomic symptoms including orthostatic hypotension [4] and Van der Eecken et al. reported cases with parkinsonism and striatonigral degeneration at autopsy in the same year. The term ‘MSA’ was first introduced in 1969 by Graham and Oppenheimer to represent all three neurological entities: olivopontocerebellar atrophy (OPCA), the Shy–Drager syndrome, and striatonigral degeneration (SND) [5]. Papp et al. first demonstrated the presence of argyrophilic glial cytoplasmic inclusions in the central nervous system of patients diagnosed with MSA in 1989 [6]. These inclusions were later reported to be positive for α-synuclein, and nowadays, alpha-synuclein inclusions in oligodendroglia are the recognized neuropathologic hallmarks of MSA and may even represent a primary pathologic event [7]. Together, Parkinson’s disease, dementia with Lewy bodies, and MSA are called α-synucleinopathies [8]. The mechanisms by which α-synuclein, a neuronal protein, ends up in the oligodendroglia causing pathology in MSA remain unclear. There is growing evidence that oligodendroglial pathology and “prion-like” spreading of misfolded α-synuclein may be the primary events in MSA leading to neurodegeneration [7–9]. In addition, proteasomal and mitochondrial dysfunction [10], dysregulation of myelin lipids [11, 12], genetic factors [13], microglial activation [14], neuroinflammation [15], proteolytic disturbance, autophagy [16], and other factors contributing to oxidative stress [17] have been reported as part of the pathogenesis of MSA.

MSA is an orphan disease [18] with an estimated incidence of 0.6–3 per 100,000 people [19, 20]. The incidence increases with age reaching 12/100,000 in those aged over 70 [21], with no difference between men and women [22, 23]. Prevalence estimates range from 1.9 to 4.9 [24] and may reach up to 7.8 per 100,000 after the age of 40 [20, 22, 23].

Given its varied clinical manifestation, MSA is frequently misdiagnosed, especially at disease onset. An autonomic presentation of MSA can be indistinguishable from pure autonomic failure (PAF). PAF is currently considered an idiopathic, sporadic, rare neurodegenerative disorder characterized by autonomic failure without other neurological symptoms or signs [25]. Recently, PAF has received more attention from researchers, partly due to shared common features with other forms of synucleinopathy. Furthermore, recent studies show that a significant proportion of patients with PAF eventually develop DLB, PD, or MSA over time. There are certain features that could predict these conversions [26, 27] raising the question of whether PAF is indeed a distinct disease entity or simply a prodromal disease stage of alpha-synucleinopathies.

A patient presenting with parkinsonism with autonomic involvement may be misdiagnosed as suffering from Parkinson’s disease. Ataxias resulting from diverse etiologies such as toxins, immune-mediated, or genetic forms—for example fragile X–associated tremor ataxia syndrome, spinocerebellar ataxia (especially type 6), or late-onset Friedreich’s ataxia [2]—can all mimic the cerebellar MSA phenotype. False-positive MSA diagnoses are also frequent. About 20% of cases diagnosed in life as MSA subsequently have a pathology confirming PD or DLB [28].

MSA is characterized by worsening of motor and non-motor features over an average of 10 years, with a fast progression from disease onset, helping to distinguish it from similar degenerative conditions, described above [29]. Approximately 50% of patients require walking aids within 3 years from the onset of motor symptoms [30]; 60% require a wheelchair after 5 years [31] with a median time to becoming bedridden of 6–8 years [30]. However, a more benign MSA variant with longer survival of over 15 years has been reported in pathology-confirmed cases [[2, 32], as well as an aggressive MSA phenotype, with a very short disease duration of less than 3 years [33]. Older age at onset [30, 31, 34–36], a parkinsonian phenotype [29, 35], early development of severe autonomic failure [30, 35, 37, 38], and severe urinary retention and nocturnal stridor [39, 40] are all negative prognostic factors, whereas a cerebellar phenotype [34] and later onset of autonomic failure [32] predict slower disease progression. Bronchopneumonia, urosepsis, and sudden death are the main causes of death in MSA. Sudden death, which often occurs at night, is thought to result from either acute bilateral vocal-cord paralysis or acute disruption of the brainstem cardiorespiratory drive.

Striatonigral degeneration (SND) and olivopontocerebellar atrophy (OPCA) are pathological subtypes of MSA. Four levels of pathological severity have been described on autopsy of MSA brains. The typical α-synuclein immunoreactive inclusion pathology (GCIs) within oligodendrocytes [41] are at the most severe end and are required for a definite postmortem diagnosis of MSA. Less frequent are neuronal cytoplasmic (NCI) and neuronal nuclear inclusions (NNI), glial nuclear (GNI), astroglial cytoplasmic inclusions, and neuronal threads, also composed of α-synuclein. The next level of severity is characterized by selective neuronal loss and axonal degeneration involving multiple regions of the nervous system with predominant involvement of the striatonigral and OPC systems, followed by myelin degeneration with pallor and reduction in myelin basic protein (MBP), with accompanying astrogliosis. The final and most mild form of pathology is microglial activation alone [17].

Glial cytoplasmic inclusions (GCIs) and the resulting neurodegeneration commonly occur across multiple CNS sites. In addition to the striatonigral and OPC involvement, CGIs are found in the autonomic nuclei of the brainstem [locus coeruleus (LC), nucleus raphe, dorsal vagal nuclei, etc.], spinal cord, sacral visceral pathways [42], and the peripheral nervous system [43], defining MSA as a truly multi-system disease [7].

Currently, MSA is considered an oligodendrogliopathy with a secondary neuronal involvement. Both a reactivation of α-syn gene (*SNCA*) [44] hypothesis and an uptake from neurons or from the extracellular environment [45, 46] have been proposed. A prion-like mechanism of propagation in MSA [47, 48] predicts the existence of “strains” with defined incubation times and patterns of neuropathology. Strains of alpha-synuclein (fibrils and ribbons) represent different conformational polymorphs of the protein. The existence of different alpha-synuclein strains may be related to different phenotypes. For example, fibrillar α-syn polymorphisms underlies different propagation patterns of α-syn pathology [49], while α-synuclein in glial cytoplasmic inclusions (GCI-α-Syn) forms more compact and about 1,000-fold more potent structures than Lewy bodies-α-Syn in seeding α-Syn aggregation, consistent with the highly aggressive MSA [50]. Also, it has been shown that oligodendrocytes, and not neurons, transform misfolded α-Syn into a GCI-like strain, emphasizing the fact that different intracellular milieus generate distinct α-Syn strains and the GCI-α-Syn maintains its high seeding activity when propagated in neurons.

The diagnostic criteria for MSA define three levels of certainty: possible, probable, and definite MSA [3] (Table 1). However, a widening range of pathological and clinical presentations have been described in MSA, including several subtypes of MSA that do not fit into the current classification (Table 2) [51].

**Table 1 D:** iagnostic criteria for possible, probable, and definite MSA

Definite MSA	Probable MSA	Possible MSA
Criteria		
Neuropathological findings during postmortem examination of: Widespread and abundant cerebral α-synuclein–positive glial cytoplasmic inclusions Neurodegenerative changes in striatonigral or olivopontocerebellar region	A sporadic, progressive, adult (> 30 years)—onset disease characterized by: Autonomic failure involving urinary incontinence (inability to control the release of urine from the bladder, with erectile dysfunction in males) or an orthostatic decrease of blood pressure within 3 min of standing of at least 30 mm Hg systolic or 15 mm Hg diastolic and Poorly levodopa-responsive parkinsonism (bradykinesia with rigidity, tremor, or postural instability) or A cerebellar syndrome (gait ataxia with cerebellar dysarthria, limb ataxia, or cerebellar oculomotor dysfunction)	A sporadic, progressive, adult (> 30 years)—onset disease characterized by: Parkinsonism (bradykinesia with rigidity, tremor, or postural instability) or A cerebellar syndrome (gait ataxia with cerebellar dysarthria, limb ataxia, or cerebellar oculomotor dysfunction) and At least one feature suggesting autonomic dysfunction (otherwise unexplained urinary urgency, frequency or incomplete bladder emptying, erectile dysfunction in males, or significant orthostatic blood pressure decline that does not meet the level required in probable MSA) and At least one of the additional features: Possible MSA-P or MSA-C Babinski sign with hyperreflexia Stridor Possible MSA-P Rapidly progressive parkinsonism Poor response to levodopa Postural instability within 3 years of motor onset Gait ataxia, cerebellar dysarthria, limb ataxia, or cerebellar oculomotor dysfunction Dysphagia within 5 years of motor onset Atrophy on MRI of putamen, middle cerebellar peduncle, pons, or cerebellum Hypometabolism on FDG-PET in putamen, brainstem, or cerebellum Possible MSA-C Parkinsonism (bradykinesia and rigidity) Atrophy on MRI of putamen, middle cerebellar peduncle, or pons Hypometabolism on FDG-PET in putamen Presynaptic nigrostriatal dopaminergic denervation on SPECT or PET

Adapted from [3]

*MSA *multiple system atrophy, *MSA-P *clinical phenotype of MSA dominant parkinsonian features, *MSA-C *clinical phenotype of MSA associated with dominant cerebellar features, *MRI *magnetic resonance imaging, *FDG-PET *fluorodeoxyglucose-positron emission tomography, *SPECT* single-photon emission computed tomography, *PET* positron emission tomography

**Table 2:** Subtypes of MSA that do not fulfill current diagnostic criteria

Type	Description
MSA with mixed pathology	Abundant α-synuclein inclusions, identified as frontotemporal lobe degeneration with α-synuclein (FTLD-synuclein) in the presence of SND and variable OPC degeneration, but in the absence of autonomic dysfunction [205]. Clinical features are typically consistent with frontotemporal dementia and a progressive non-fluent aphasia with no autonomic dysfunction
Non-motor variant MSA	A non-motor variant of pathologically confirmed MSA showing neither parkinsonism nor cerebellar symptoms [195]. The case presented clinically with 10-year history of autonomic failure (orthostatic hypotension and neurogenic bladder), RBD and a 2-year history of stridor prior to sudden death
“Benign” MSA	Described as cases with prolonged survival up to 15 years or more in 2–3% of MSA patients [152]. Most of them showed a slowly progressing parkinsonism resembling PD in the first 10 years of disease with subsequent rapid deterioration after the development of autonomic failure, before which a correct diagnosis was difficult. Many of these patients developed motor fluctuations and levodopa-induced choreiform dyskinesias leading to consideration of deep brain stimulation, which is a procedure currently not recommended for MSA patients [199]. These rare, long-surviving patients with MSA-P, were considered “benign” forms [32]. Pathological examination of patients with survival longer than 18 years has revealed an extensive distribution of GCIs in the CNS [206]
“Incidental MSA”	The presence of GCIs may represent an age-related phenomenon not necessarily processing to overt clinical disease similar to incidental Lewy body disease [207]
“Minimal change” MSA-P	A rare aggressive form with GCIs and neurodegeneration was almost completely restricted to the SN and putamen, thus representing “pure” SND [208]

*FTLD *synuclein-frontotemporal lobar degeneration, *SND* striatonigral degeneration, *OPC* olivopontocerebellar, *RBD* REM sleep behavior disorder, *PD* Parkinson disease, *MSA *multiple system atrophy, *MSA-P *clinical phenotype of MSA dominant parkinsonian features, *GCIs* glial cytoplasmic inclusions, *CNS *central nervous system, *SND *striatonigral degeneration

Based on the predominant clinical phenotype, MSA is categorized into MSA-P when parkinsonism is predominant and is associated with SND, and MSA-C, with OPCA, when associated with dominant cerebellar features. The combination of both forms, “mixed” MSA, also exists [3]. In Asian populations, the majority of MSA cases—about 70–80% [52]—are of MSA-C type, whereas in the Caucasian population, MSA-P type predominates (about 67–84%) [29, 53]. MSA-P is characterized by parkinsonism with rigidity, postural instability with a tendency to fall, and poor response to levodopa [54]. The motor symptoms are usually symmetrical [55]. Rest tremor is rare, whereas irregular postural and action tremor may occur [57]. MSA-C is associated with cerebellar ataxia affecting arms, legs, action tremor, downbeat nystagmus, and hypometric saccades [56]. Hyperreflexia and Babinski sign may occur in 30–50% of patients, while abnormal postures, such as bent spine, antecollis, and hand or foot dystonia, are rare [56]. Dysphonia, repeated falls, drooling, dysphagia, dystonia, and pain occur in advanced stages of the disease [57]. Spinal myoclonus in MSA-C caused by α-synuclein deposition in the spinal cord has also been reported [58].

The onset of motor symptoms is on average around 56 years of age (± 9 years), with both sexes equally affected [59]. However, up to 75% of MSA cases have a prodromal phase with non-motor symptoms, such as cardiovascular autonomic failure, orthostatic hypotension, urogenital and sexual dysfunction, REM-sleep behavior disorder, and respiratory disorders. These may precede the motor presentation by months to years [60]. Furthermore, up to 95% of MSA patients experience non-motor symptoms at some point during the course of the disease with autonomic failure, in particular urogenital (urinary incontinence and impaired detrusor muscle contractibility) and cardiovascular symptoms being frequent and early features of MSA [61].

In this review, we describe this wide clinical spectrum of features now seen in MSA with a focus on the non-motor symptoms and the premotor phase, and provide an up-to-date overview of the current understanding in this fast-growing field.

## The neuropathology of non-motor features in MSA

The distribution of neurodegenerative changes in patients with MSA is broadly reflected by α-synuclein-positive oligodendroglial cytoplasmic inclusions [2]. During the course of the disease, most patients present with a combination of gastrointestinal, cardiovascular, urogenital, or thermoregulatory abnormalities of different severities [2, 36]. Several brain regions of MSA patients are severely depleted of dopamine and norepinephrine including the corpus striatum, nucleus accumbens, substantia nigra, locus coeruleus, hypothalamus, and septal nuclei [62]. A severe loss of A5 noradrenergic neurons in the pontine tegmentum has been shown in MSA, leading to respiratory and cardiovascular manifestations, along with similar severe losses of noradrenergic neurons in the locus ceruleus [63, 64]. The loss of C1-group neurons (neurons that synthesize epinephrine) contributes to orthostatic hypotension in MSA, with the severity of neuronal loss in MSA being greater than in PD and DLB [65, 66]. Impaired reflex release of vasopressin in response to hypotension or hypovolemia in MSA may be a consequence of degeneration of noradrenergic projections to the magnocellular vasopressin neurons from A1 neurons, localized in the caudal ventrolateral medulla [65, 67]. Reduced orexin immunoreactivity, likely associated with sleep apnea syndrome, has been observed in the nucleus basalis of Meynert in MSA patients [68]. Tyrosine hydroxylase neuronal loss in the periaqueductal gray of MSA, similar to Lewy body disease, has also been reported [69].

Involvement of the insular cortex has been related to non-motor symptoms in MSA, including autonomic dysfunction and arousal [70]. Activation of the subgenual and pregenual portions has been associated with cardiovagal responses in normal people; activation of the dorsal anterior cingulate cortex (the midcingulate cortex), part of the salience network, has been shown to be associated with sympathetic activation related to behavioral arousal [71].

The brainstem regions, in particular those where the respiratory centers are located, are frequently affected in MSA. The ventral medullary arcuate nucleus, likely responsible for respiratory chemosensitivity, degenerates in MSA [72]. In addition, the pre-Botzinger complex of the medulla and the neurokinin-1 immunoreactive neurons, responsible for respiratory rhythmogenesis, are similarly markedly affected [73, 74]. The neuronal loss in MSA, from the dorsal motor nucleus of the vagus (DMV), located in the dorsomedial medulla [75], may contribute to baroreflex-triggered cardioinhibition and a respiratory sinus arrhythmia [76].

REM-sleep behavior disorder (RBD) is commonly seen in this disease and may relate to pathology affecting the pontomedullary brainstem nuclei in MSA [77, 78]. During REM sleep, atonia is controlled by the pontomedullary structures [the magnocellular reticular formation (MCRF), sublaterodorsal (SLD), and pedunculopontine and laterodorsal (PPN/LDT) nuclei] [77]. Elimination of atonia during REM sleep, caused by lesions in SLD/SC and MCRF (even unilaterally), likely leads to dream enacting behavior [79]. Depletion of cholinergic neurons in the PPN/LDN complex has been shown in autopsy studies of patients with MSA [80]. PPN may have a modulatory role in REM-related phenomena rather than a primary role for the atonia during REM sleep [75].

Preganglionic and postganglionic sudomotor denervation probably underlies the anhidrosis seen in MSA, with more postganglionic dysfunction seen later in the disease [76]. Postganglionic cardiac sympathetic denervation also has been shown to occur in a minority of cases of MSA[81}. The preganglionic neurons innervating the detrusor muscle [82] and loss of neurons of the Onuf nucleus innervating the sphincter are related to early and severe bladder dysfunction.

## Clinical manifestations of non-motor features

### Cardiovascular failure in MSA

#### Orthostatic hypotension

Early symptoms in MSA are frequently related to cardiovascular and autonomic failure and precede motor manifestations. In a recent study, the majority of MSA patients (77%) recalled the early autonomic symptoms, initially not recognized as being a manifestation of a neurodegenerative disease [83].

Criteria for diagnosis of probable MSA according to Gilman et al. [3, 84] involve autonomic dysfunction including urinary incontinence (inability to control the release of urine from the bladder, with erectile dysfunction in males) or orthostatic hypotension (OH) (decrease of blood pressure within 3 min of standing by at least 30 mmHg systolic or 15 mmHg diastolic).

Importantly, autonomic dysfunction may be the only presenting feature in some MSA patients [85]. Symptomatic OH, the main symptom of cardiovascular autonomic failure, often manifests as recurrent syncope, dizziness, nausea, headache, and weakness, and has been reported in 43–81% of all MSA patients [86, 87]. Cardiovascular autonomic failure associated with degeneration of the nucleus ambiguus has been reproduced in an MSA mouse model [88].

An important differential for the autonomic failure in the early stage of MSA is the diagnosis of pure autonomic failure (PAF). Orthostatic hypotension (OH) and its related symptoms, such as light-headedness or dizziness, and sudomotor dysfunction, are the most common presenting feature in PAF [89–91]. Bladder symptoms are also present in some PAF patients, but these features are less severe than in MSA [92]. While respiratory symptoms such as stridor or sighing are common in MSA, these symptoms were not reported in patients with PAF [93]. There are only a few studies that have reported pathology in patients with PAF. Intracytoplasmic eosinophilic inclusions with Lewy bodies resembling those in PD patients were reported in the neurons in autonomic ganglia and postganglionic nerves [91]. Although typical PAF patients have no parkinsonian features, Lewy body deposition in the substantia nigra, locus coeruleus, thoracolumbar, and sacral spinal cord with limited neuronal loss has been described in a case report [90]. Later, pathologically confirmed studies also demonstrated pre- and postganglionic autonomic lesions in PAF patients [91, 94]. These findings suggest that PAF is a limited and peripheral form of alpha-synucleinopathy, in contrast to more central forms in PD and DLB [95].

PAF patients have hyposmia, reduced cardiac MIBG reuptake, and Lewy body deposition both in the skin and central nervous system, similar to PD [96–99]. There is increasing awareness of the progression of patients with the PAF phenotype to other alpha-synucleinopathies. In one study, within 4 years of onset, 34% of patients with PAF phenoconverted to a manifest CNS alpha-synucleinopathy, either DLB (18%), PD (8%), or MSA (8%). The prognostic implications of autonomic failure are unknown and predictors of phenoconversion to MSA have yet to be determined [27, 100]. However, it took on average 5 years for patients with PAF to develop motor deficits leading to the diagnosis of MSA, and nearly twice as long (9.5 years) to diagnose PD or DLB. Compared to patients who phenoconverted to PD or DLB, onset of symptomatic neurogenic orthostatic hypotension in MSA patients occurred at a younger age (≈ 52 years), with a shorter median time to diagnosis (≈ 5 years) [27]. The presence of RBD and preserved olfaction in patients with PAF increased the probability of a future diagnosis of MSA, whereas olfactory loss increased the odds of developing PD/DLB [27]. These findings highlight the need for more research in identifying biomarkers that could be useful in aiding earlier diagnosis.

#### Supine hypertension

Supine hypertension occurs in ~ 50% of patients with autonomic failure and orthostatic hypotension, as has been observed in MSA [101]. This may be a problem at night if such patients lie supine and horizontal as there is often a reversal of the circadian change in blood pressure [102]. A recent study also described the occurrence of abnormal circadian BP rhythm (absent nocturnal fall or a reversed nocturnal fall of blood pressure) in about half of the patients with PD. However, this feature and a reversed nocturnal circadian blood pressure rhythm was found to be much more common in MSA compared to idiopathic PD. This finding suggests that autonomic dysfunction plays an important contributing role in the control of circadian BP rhythms [103].

#### Postprandial hypotension (PPH)

Food ingestion can lower systemic blood pressure in MSA but not in healthy individuals, who have normal compensatory cardiac and regional haemodynamic responses. PPH is also an important feature of autonomic failure in patients with MSA [104]. PPH is present in both MSA-P and MSA-C, but the severity in MSA-C is greater than those with MSA-P [105]. PPH is also common in PD, even in those without other features of autonomic failure [106].

#### Exercise-induced hypotension

Exercise-induced hypotension has been reported in MSA [107]. An increase in sympathetic activity to the vasculature of non-exercising muscles and the other organs plays a major role in maintaining blood pressure. The consequences of exercise-induced hypotension can occur at various stages of exercise. Exercise-induced hypotension in those with MSA-C has been found to be greater than those with MSA-P in the supine position, but this does not get worse when the MSA-C patient is in the orthostatic position [108].

### Bladder dysfunction in MSA

A recent study showed that 18.2% of MSA patients presented with bladder dysfunction as the initial manifestation, on average 2.8 years before any motor manifestation [109]. In men, urinary symptoms are typically preceded by erectile dysfunction. Bladder dysfunction, particularly urinary retention in MSA, may reflect pathology in the sacral spinal cord, a common site for MSA pathology. These findings are important especially for patient care. Surgical treatment of bladder outlet obstruction should be avoided, as it often fails in MSA patients, whereas prostatic surgery is not contraindicated in patients with PD.

Three uro-neurological features are important in MSA. These include large post-void residual urine volumes of > 100 ml without prostatic hyperplasia in men and without common neurologic conditions (lumbar spondylosis and diabetes) [110, 111], an open bladder neck during filling-phase videourodynamics [112, 113], which is not uncommon in stress-incontinent women, but is extremely rare in men, and a sphincter electromyography (EMG) abnormality—sphincter denervation [112]. These are explained by the neuronal cell loss in Onuf’s nucleus (a group of anterior horn cells found in the sacral spinal cord), which seem to degenerate early in the course of MSA [114]. Electrophysiologically, this leads to prolonged polyphasic sphincteric motor unit potentials (MUPs) on anal sphincter EMG testing. Although sphincter EMG abnormalities are noted in some patients with PD, dementia with Lewy bodies, pure autonomic failure, and progressive supranuclear palsy, this test still has an important diagnostic value in MSA especially when viewed in the context of other clinical features and paraclinical investigations [112].

### Sleep disorders in MSA

MSA patients often present with nighttime sleep problems including rapid eye movement (REM) sleep behavior disorder (RBD) [115], periodic limb movements (PLMs), restless legs syndrome (RLS), or RLS-like symptoms [116], and all lead to sleep fragmentation and decreased sleep efficiency. However, this aspect of MSA has only been investigated in small cohorts with even less data comparing the polysomnographic (PSG) parameters. [117]. One study showed similar nighttime problems and PSG results for both MSA-P and MSA-C patients, despite different lesion localization of the pathologic processes in the two MSA subtypes [117]. Polysomnographic studies reported that in MSA patients, the total sleep time and sleep efficiency is reduced and waking after sleep onset is increased [118].

Rapid eye movement sleep behavior disorder (RBD) is the most common sleep-related disorder in MSA [119] and may precede the development of parkinsonism by years [120]. RBD is characterized by vigorous and injurious behaviors related to vivid, action-filled, and violent dreams during nocturnal REM sleep and REM sleep without atonia (RWA) [121] as determined by a polysomnogram (PSG) [115]. In postmortem studies of patients with RBD, the most frequent finding is neurodegeneration with abnormal deposition of α-synuclein in pontomedullary brainstem nuclei [77].

The prevalence of RBD, as determined by polysomnography, is very high (88%) in MSA patients. RBD signs have been found to be present in about half of MSA patients before the onset of motor symptoms and are present equally in both in MSA-P (82%) and MSA-C (83%) patients [122], suggesting that RBD may represent a prodromal phase in MSA [123]. However, RBD is not restricted to the prodromal phase, as up to 30% of MSA patients developed RBD before, 7% at the same time, and 63% after the onset of motor symptoms of MSA [124]. Generally, patients with idiopathic RBD are predominantly male [115]. However, patients with MSA having RBD are not predominantly male [125], similar to that seen with PD patients and RBD [126].

Interestingly, motor control has been shown to be transiently improved during RBD in patients with MSA, as confirmed by video recordings. Up to 80% of patients exhibited improvement in their movements, speech, or facial expression during REM sleep compared to the awake state. The improvement was present even in the most affected patients. There was no difference in the percentage of REM-related motor improvement between patients who were affected with MSA-P or MSA-C. However, the rate of motor or vocal improvement during REM sleep was 30–60% greater in PD than in patients with MSA, except for hypomimia [124].

Usually, RBD symptoms in MSA patients exist for a limited period shortly before the onset of other neurological symptoms, but generally disappear within a few years of the onset of neurological symptoms. This is likely due to progressive degeneration of the neuronal structures in the brainstem responsible for the occurrence of RBD [125].

### Respiratory features in MSA

Respiratory problems, including stridor, sleep-disordered breathing (e.g., obstructive sleep apnoea—OSA) and respiratory insufficiency, are known to occur in MSA [118]. Of these, stridor is part of the second consensus diagnostic criteria [3] and sleep apnea represents a major cause of death in MSA [127, 128]. Nocturnal stridor and obstructive sleep apnea are the most common sleep-related breathing disorders in MSA patients [129].

OSA affects between 15 and 37% of MSA patients [118]; nocturnal stridor (a strained, high-pitched, harsh respiratory sound) varies from 13 to 69% [130]. In one study, stridor was more frequently detected in MSA-C than in MSA-P patients [118], but the opposite has also been seen [129]. Stridor is associated with a poor prognosis in MSA. It is unclear whether death results from the vocal-cord paralysis per se, central hypoventilation, or both [64].

The causes that lead to nocturnal stridor are related to upper airway obstruction, such as vocal-cord abductor paralysis; an impairment of the respiratory center, such as Cheyne–Stokes respiration; or an impaired hypoxemic ventilatory response [131]. Laryngeal electromyography in patients with MSA and autonomic failure has revealed the evidence of denervation of the posterior and interarytenoid cricoarytenoid and partial denervation in the cricopharyngeal sphincter, sometimes leading to respiratory obstruction requiring a tracheostomy.

Vocal-cord paralysis can also occur in MSA. A history of nighttime snoring is usually the first sign; importantly, this is not oropharyngeal snoring, but laryngeal stridor and video polysomnography can differentiate between the two by examining the respiratory phases, whether inspiratory, expiratory, or both [132]. Bilateral vocal-cord paresis frequently occurs in MSA and can sometimes lead to death. Patients with symptoms of airway obstruction need a tracheostomy to bypass the level of obstruction. These tracheostomies are rarely removed because of the progressive nature of the disease [133].

In some MSA cases, hypertonicity rather than paresis of the vocal cords has been described using direct electromyography [134]. During episodes of stridor, the cords adopt a paramedian position, best diagnosed by a combination of laryngeal electromyography with a response to botulinum toxin being used to distinguish hypertonicity from paralysis in some cases.

Patients with MSA have impaired ventilatory drive, with minimal-to-no chemosensitivity to hypoxia [137]; thus, they may be at a significantly higher risk of becoming hypoxic without any reflex capacity to compensate. In addition, patients with MSA often have disturbances of respiratory rhythm during sleep [138]. These may explain why patients with MSA may die of respiratory insufficiency despite tracheostomy [41]. Clearly, some MSA patients have central hypoventilation in addition to stridor, which can only be diagnosed during repeated sleep evaluations.

### Cognitive involvement in MSA

Cognitive impairment is an integral part of MSA, although dementia is a non-supporting diagnostic feature [3, 137]. In autopsy-confirmed MSA, the prevalence rates of mild, moderate, and severe cognitive impairment have been reported to be 22%, 2%, and 0.5%, respectively [137, 138].

Executive dysfunction is a prominent cognitive problem in MSA, affecting up to half of the patients [139–141]. This includes problems with semantic and phonemic word list generation [142, 143], perseverative behavior [144], and diverse impairments of problem solving, response inhibition, flexibility, attention, and working memory [142, 144].

Memory disturbances not reaching a diagnosis of dementia were observed in up to 66% of MSA patients. They were characterized by impaired learning [141], immediate [[145] and delayed recall, [139–141] and less often, impaired recognition [139], visuospatial and constructional difficulties [140, 143, 145].

On average, it takes about 7 years from MSA diagnosis for clinically significant cognitive symptoms to develop [[35], but pathologically proven MSA cases with earlier cognitive impairment [146–148] have been reported. Among MSA patients surviving more than 8 years, almost half of them develop cognitive problems [139], suggesting that the cumulative prevalence of dementia in MSA would be similar to that of Parkinson’s disease (PD), if the disease had a longer course [149, 150]. Furthermore, 14% of MSA patients have been found to have severe cognitive impairment in the year before death [35] and exceptionally long-term MSA survivors showed dementia onset between 13.5 and 17 years from the onset of the disease [32].

Cognitive impairment in MSA is predicted by factors such as greater motor disability [139, 145, 151, 152], male gender, educated for less than 10 years and the presence of early cardiovascular dysautonomia [153].

Cognitive impairment and MSA clinical subtypes have produced controversial results [141, 143, 154] with some reports, suggesting that the MSA-P patients have more problems with visuospatial and constructional function, verbal fluency, and executive functions [143, 153], while the MSA-C patients were impaired only in visuospatial and constructional functions [153]. These data suggested a more severe and widespread impairment in MSA-P patients despite similar disease duration.

Furthermore, cognitive deficits in MSA-C have been linked to degeneration in cerebral cortex, the pontocerebellar system, and the disruption of cerebrocerebellar connections (cerebrocerebellar circuitry) [155, 156]. Described as reciprocal cerebrocerebellar circuits [157], the so-called cerebellar cognitive affective syndrome was mainly observed in acute cerebellar diseases such as infarctions and tumors. This syndrome is characterized by executive dysfunction, impaired spatial cognition, including visuospatial disorganization, and impaired visuospatial memory. Deficients in visuospatial function in this cerebellar syndrome give support to the hypothesis of a cerebellar contribution cognitive impairment in MSA. However, other investigators failed to demonstrate the contribution of cerebellar dysfunction in the cognitive profile of MSA patients [155].

Progressive frontotemporal degeneration on neuroimaging [140, 158–161] and postmortem findings of neuronal loss, astrogliosis, and GCI accumulation in frontal and temporal regions of MSA patients with dementia, further point towards cognitive decline as a characteristic feature in some MSA patients [137].

Almost all MRI studies have shown that there is a characteristic pattern of prefrontal, frontal, temporal, and parietal cortical atrophy in MSA-P [161–164] and MSA-C [165–168], although some qualitative differences between subgroups have been reported [168]. The distribution of cortical atrophy is in line with the findings of hypometabolism on fluorodeoxyglucose (FDG) positron emission tomography (PET) in prefrontal and frontal [169, 170], temporal, and parietal regions in MSA-P [170] and in frontal and inferior parietal regions in MSA-C [171, 172]. In favor of the hypothesis that there is a primary subcortical deafferentation of cortical regions, a correlation between pontine, midbrain, and cerebellar atrophy and impairment in different cognitive domains as well as global cognition in MSA patients has been reported [160], supported by the observation of cerebellar hypoperfusion in association with visuospatial decline in MSA-C [143]. Conversely, prefrontal atrophy has been shown to correlate with overall memory scores in MSA as a group [154], and in addition, there is a correlation between dorsolateral prefrontal hypoperfusion and visuospatial impairment in both motor MSA subtypes and executive dysfunction in MSA-P, all of which supports a primary cortical pathology in this aspect of MSA symptomatology [143].

PET and neuropsychological examination in MSA patients with different disease duration found that patients with a shorter duration showed executive and verbal memory dysfunctions, while patients with longer duration had multiple-domain cognitive impairment, including visuospatial deficits [140].

### Emotional lability and depression in MSA

Patients with MSA frequently report emotional lability, with short (sometimes only one or two minute) episodes of crying due to happiness, or sadness, in response to relatively minor environmental stimuli. Pathological laughter and crying (PLC) is a condition in which a patient with an underlying neurological disorder exhibits episodes of laughter or crying or both, without an apparent motivating stimulus or to a stimulus that would not have elicited such response in the past. This problem is characterized by a deficit in the regulation and coordination of emotional expression [173]. PCL can provoke restrictive life-style modifications and even lead to social isolation. Despite its high prevalence and quality of life implications, it remains surprisingly understudied, and in clinical settings, it is often unrecognized [174]. Several scales are available to identify and characterize PLC. Among these, the two most commonly used in clinical research studies are the Pathological Laughter and Crying Scale and the Center for Neurologic Study-Lability Scale [175].

Importantly, PLC and mood disorders may coexist in up to half of MSA patients [175]. The resolution of pathological laughing or crying seems to be independent of the changes in anxiety or aggression scores [175]. A previous study found that all patients with MSA who exhibited pathological laughter and/or crying also had depression [176].

40–85% of patients with MSA display mild depression [141, 144, 153, 177–179], and 1/3 are moderately-to-severely depressed [177, 180]. Anxiety is reported to affect about 37% of MSA patients [143, 181]. Both MSA motor subtypes are associated with high levels of depression and anxiety [141, 143, 154, 180, 182], with some studies, suggesting that MSA-P patients are more depressed, while MSA-C subjects are more anxious [154, 182]

Psychosis in MSA has also reported in some cases and up to 9.5% of patients with MSA-P develop hallucinations [183].

### Other non-motor features in MSA

Gastrointestinal symptoms are frequent complaints in MSA resulting from reduced gastrointestinal motility due to autonomic nervous system dysfunction with delay in gastric emptying [184] occurring at the early stages of the disease [61, 93]. Constipation is a well-known feature of MSA and can predate the onset of typical motor symptoms. Constipation may be explained by prolonged colonic transit time in line with a reduction of phasic rectal contraction and abdominal strain in patients with MSA [185]. A change in bowel habits suggests autonomic involvement of the lower gut [186].

Other non-motor features include vasomotor failure with diminished sweating (hypohidrosis) [187] and pupillomotor abnormalities. Usually, visual acuity, colour vision, and visual fields remain unaffected [188]. Sweating disorders, mainly manifesting as widespread anhidrosis, frequently occur in patients with MSA [39, 189]. The postganglionic sympathetic sudomotor involvement is only partly responsible for sudomotor dysfunction and suggests the involvement of postganglionic fibres or sweat glands later in the disease course [76]. Widespread progressive anhidrosis is a more severe feature in MSA compared to that seen in PD and PSP [190, 191]. Pupil abnormalities are not commonly recognized as features of MSA, although these have occasionally been reported including in the original cases of Shy and Drager’s patients [4]. Pupillary response to eye drops was recently proposed as a useful aid for distinguishing PD and MSA [192]. However, the pupillary measurement in these disorders needs further study to confirm the usefulness of these proposed diagnostic tests.

Autonomic dysfunction was initially believed to be solely associated with preganglionic abnormalities in the brainstem and spinal cord in MSA [39, 193]. However, evidence of postganglionic involvement has been reported by showing that up to 30% of MSA patients have reduced (123)I-metaiodobenzylguanidine (MIBG) uptake at all stages of disease [194]. Furthermore, sudomotor abnormalities including impairment at pre- and postganglionic sites may coexist, indicating a further progression over time and the involvement of postganglionic fibres or sweat glands later in the disease course [76].

## The premotor phase in MSA

The prodromal non-motor phase of MSA often precedes the motor symptoms by several months to years. Reports of pathologically proven MSA cases in which no classic motor symptoms (cerebellar, parkinsonian, or pyramidal tract) developed during the disease course [195] emphasize the need to consider premotor symptoms in the early evolution of the disorder. Early symptoms in MSA are frequently autonomic in nature (in 73%) [83]. In men, erectile failure typically occurs 4.2 (± 2.6) years prior to diagnosis. After erectile failure, orthostatic hypotension—a consistent hallmark of MSA [191]—fatigue following exercise, urinary urgency or hesitancy, and violent dream enactment behavior consistent with REM behavioral sleep disorder, are the most frequent initial symptoms. For most patients, the onset of motor symptoms is 1 year after the onset of autonomic symptoms. Although challenging to interpret, these symptoms can provide a window of opportunity for disease-modifying interventions if recognized in time, therefore, raising a suspicion of MSA diagnosis.

The presence of autonomic failure, erectile dysfunction, urinary urgency, nocturia, sleep problems (RBD), postural dizziness, and respiratory disturbances at presentation, in the absence of ataxia and parkinsonism, should be regarded as suggestive of premotor MSA [196]. Up to half of patients later diagnosed with MSA initially were classified as having pure autonomic failure, as they did not exhibit motor symptoms at the first presentation to a doctor [138, 179].

**Fig. 1:**
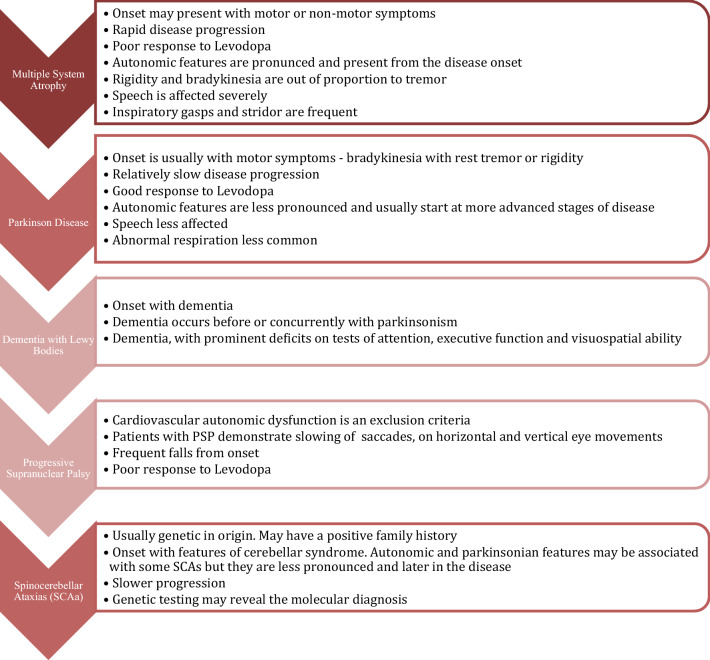
Differential diagnosis in MSA

A pattern is emerging in MSA; with non-motor symptoms preceding the classic motor features showing a distinctive pattern of early genitourinary dysfunction followed by orthostatic hypotension in association with sleep disorders such as RBD, sleep apnoea, excessive snoring, and stridor [196]. Above all, respiratory dysfunction is the most suggestive symptom of MSA in the presence of other premotor symptoms. At the same time, in the premotor phase, olfaction and cognition [197] remain relatively intact compared with other parkinsonian disorders (Fig. 1).

The diagnostic challenge in the premotor phase of MSA is the substantial overlap with the non-motor symptoms of other neurodegenerative disorders, in particular Parkinson’s disease and pure autonomic failure. However, at the time of diagnosis, autonomic dysfunction in MSA is typically more severe compared to that in PD and autonomic dysfunction preceding the development of parkinsonism supports MSA as a more likely diagnosis [138]. Conversely, a case with parkinsonism or cerebellar ataxia in the absence of erectile dysfunction or autonomic failure would make an MSA diagnosis less likely [83].

**Fig. 2:**
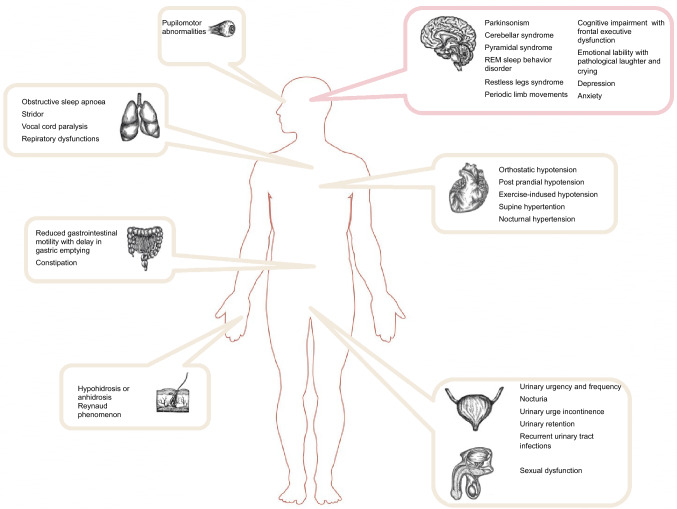
Clinical spectrum of multi-organ involvement in multiple system atrophy

The spectrum of premotor symptoms of MSA is broad (Fig. 2) and should, therefore, be investigated more vigorously to achieve an early diagnosis of this serious illness and to avoid unnecessary therapeutic interventions [196].

## Treatment of non-motor symptoms in MSA

Symptomatic treatment remains the current standard of care in MSA. Management strategies are focused on symptom control with medication for parkinsonism, autonomic lability, bladder and bowel dysfunction, and mood problems [198] (Table 3). Deep brain stimulation is not recommended for MSA [199].

**Table 3:** Symptomatic treatment in MSA

Symptoms	Pharmacological treatment	Nonpharmacological treatment
Non-motor symptoms		
Orthostatic hypotension Postprandial hypotension Supine hypertension	Ephedrine Fludrocortisone (needs to be avoided if patient also has supine hypertension) Midodrine L-threo-DOPS Octreotide Atomoxetine (avoid before bedtime) Droxidopa Nebivolol Clonidine Sildenafil at bedtime Nitroglycerine patch at bedtime Losartan Eplerenone Nifedipine Avoid before bedtime—non-steroidal anti-inflammatory drugs (ibuprofen and indomethacin), norepinephrine transporter inhibitors (atomoxetine)	Elastic stockings Adequate and higher salt and fluid intake Head-up tilt during the night Smaller but more frequent meals Special set of exercises Limit drinking water 60–90 min. before bedtime Tilt the whole bed head-up by approximately 10-20º, in patient not tolerating this measure tilt only head of the bed up to 30º Carbohydrate-rich snack at bedtime
Urinary dysfunction Urinary incontinence Incomplete bladder emptying Nocturia	Oxybutynin Injections of Botulinum toxin A into the detrusor muscle Moxisylyte Prazosin Desmopressin intranasal spray	Intermittent or permanent urethral or suprapubic catheterization if post-void residual volume is > 100 ml
Erectile dysfunction	Sildenafil	
Neuropsychiatric manifestations Depression	Selective Serotonin Reuptake Inhibitors (SSRIs)	Psychotherapy
Sleep disorders REM-sleep behavior disorder	Clonazepam Melatonin Gabapentin Pregabalin Sodium oxybate Zopiclone Temazepam	
Nocturnal stridor Breathing problems		Non-invasive positive pressure ventilation (NPPV) Continuous positive airway pressure (CPAP) Tracheostomy
Motor symptoms		
Parkinsonism Cerebellar ataxia Dystonia	Levodopa Amantadine Botulinum toxin A	Physiotherapy Occupational therapy Speech therapy Physiotherapy Occupational therapy Speech therapy

Adapted from [198, 209, 210]

New strategies targeting α-synuclein are in progress [54, 200]. Unfortunately, most clinical trials have failed to show positive results due in part to the small number of enrolled patients, the inevitable accidental recruitment of non-MSA patients, and the relatively late intervention for disease-modifying treatments [201]. The only clinical trial showing positive results was intra-arterial and intravenous injection of autologous mesenchymal stem cells (MSCs), which delayed disease progression as measured by the unified MSA rating scale in patients with MSA-C [202]. Targeting the ‘prion-like’ cell-to-cell propagation of α-synuclein using an α-synuclein immunotherapy is an appealing approach and has been studied in animal models of MSA [203]. A combination of a single-chain antibody (CD5-D5) and anti-inflammatory treatment (lenalidomide) ameliorated gliosis, α-synuclein accumulation, and behavioral deficits in MBP-α-synuclein transgenic mice [204].

## Conclusion

The clinical spectrum of MSA and, especially, the non-motor features at disease onset represent a growing challenge for clinicians, researchers, and drug trials. Besides the current diagnostic criteria, several MSA subtypes, not fully fitting the currently used diagnostic criteria, are emerging, highlighting the complexity of the disease as well as the need for revised diagnostic tools. The ability to make an earlier diagnosis and thus intervention in MSA may lead to an increased quality of life, a better prognosis, as well as a greater chance of finding disease-modifying drugs.
